# Hepcidin-25, Mean Corpuscular Volume, and Ferritin as Predictors of Response to Oral Iron Supplementation in Hemodialysis Patients

**DOI:** 10.3390/nu7010103

**Published:** 2014-12-29

**Authors:** Kazuya Takasawa, Chikako Takaeda, Teiryo Maeda, Norishi Ueda

**Affiliations:** 1 Renal Division, Public Central Hospital of Matto Ishikawa, 3-8 Kuramitsu, Hakusan, Ishikawa 924-8588, Japan; E-Mail: takaeda@imcc-med.com; 2 Biomarker Society, 1-403 Kosugi, Nakahara, Kawasaki, Kanagawa 211-0063, Japan; E-Mail: dr.tanuki@maeda-irr.com; 3 Department of Pediatrics, Public Central Hospital of Matto Ishikawa, 3-8 Kuramitsu, Hakusan, Ishikawa 924-8588, Japan

**Keywords:** anemia, ferritin, hemodialysis, hepcidin, iron, mean corpuscular volume

## Abstract

The benefit of oral iron therapy (OIT) and factors predictive of OIT response are not established in hemodialysis (HD) patients with iron deficiency anemia (IDA). We examined the values of hepcidin-25, mean corpuscular volume (MCV), and ferritin as predictors of OIT response. Oral ferrous fumarate (50 mg/day, 8 weeks) was given to 51 HD patients with IDA (hemoglobin (Hb) < 12 g/dL, ferritin < 100 ng/mL) treated with an erythropoietin activator. Sixteen patients were responders (improvement of Hb (ΔHb) ≥ 2 g/dL) and 35 were non-responders (ΔHb < 2g/dL). Baseline Hb, MCV, serum hepcidin-25, ferritin, iron parameters, and C-reactive protein (CRP) before and ΔHb after OIT were compared between groups. Hepcidin-25, MCV, ferritin, and transferrin saturation were lower in the responders than in the non-responders. Hepcidin-25 positively correlated with ferritin. Hepcidin-25, MCV, and ferritin positively correlated with baseline Hb and negatively correlated with ΔHb. Despite normal CRP levels in all patients, CRP correlated positively with hepcidin-25 and ferritin. Stepwise multiple linear regression analysis and receiver operating characteristics curve analysis revealed that hepcidin-25, MCV, and ferritin could predict OIT response. We conclude that hepcidin-25, MCV, and ferritin could be useful markers of iron storage status and may help predict OIT response in HD patients.

## 1. Introduction

Iron deficiency anemia (IDA) is an important and frequently encountered problem in patients undergoing maintenance hemodialysis (HD). Intravenous iron therapy is considered to have a superior benefit over oral iron therapy (OIT) for the management of IDA in HD patients [1,2]. However, it is controversial whether or not intravenous iron supplementation is superior to OIT in patients with chronic kidney disease (CKD) not on dialysis [3]. In addition, approximately 20% of HD patients with IDA have experienced benefits from OIT [2]. Recent studies have shown the benefit of OIT to be equivalent to intravenous iron therapy in HD patients with IDA [4,5]. OIT is inexpensive and associated with less serious adverse effects than intravenous iron therapy [1]. Thus, it is important to determine whether OIT is truly beneficial for treating IDA in HD patients. Furthermore, if OIT is beneficial, it is important to identify factors predictive of response to OIT.

IDA is characterized by low levels of hemoglobin (Hb), serum ferritin, a major biomarker of iron storage status [6,7,8], and microcytic erythrocytes with low mean corpuscular volume (MCV), resulting from defects in heme synthesis or iron availability for erythropoiesis [9]. Thus, serum ferritin and MCV have been used as biomarkers of iron deficiency [6,7,8].

Hepcidin-25 is produced by the liver and is a major regulator of iron metabolism [7,9,10]. Similarly, iron storage status can regulate hepcidin-25 and *vice versa* [7,9,10]. Production of hepcidin-25 is decreased in response to iron deficiency, whereas it is enhanced in response to increased plasma and intracellular iron stores. Increased levels of hepcidin-25 by iron loading subsequently generate a negative feedback loop to maintain a systemic balance of iron, as increased levels of hepcidin-25 inhibit iron absorption from the small intestine and iron efflux from macrophages or hepatocytes [7,9,10]. In contrast, a decrease in the levels of hepcidin-25 associated with iron depletion can enhance intestinal iron absorption and efflux from macrophages or hepatocytes, leading to increased iron availability for erythropoiesis [7,9,10]. This feedback regulation of hepcidin-25 expression by iron storage status serves to modulate iron absorption and efflux to meet the body’s iron demand.

Thus, hepcidin-25 plays a crucial role in the regulation of iron metabolism in chronic disease, including CKD [7,10]. In patients with HD or CKD, serum hepcidin-25 has been shown to be up-regulated by inflammation [11] and iron loading [12]. In fact, serum levels of hepcidin-25 are generally increased in patients with HD [13,14,15,16] or those with CKD not on dialysis [11,17], although one study reported no change in hepcidin-25 in CKD patients [18]. On the other hand, serum levels of hepcidin-25 have been shown to be down-regulated by iron depletion in patients with HD or CKD [11,19]. In addition, serum levels of hepcidin-25 have been shown to be positively correlated with serum levels of ferritin in patients with HD or CKD [13,14,15,16,17,18] and in those with IDA but no kidney disease [20], suggesting that serum levels of hepcidin-25 may predict iron storage status.

However, it remains to be determined whether or not serum hepcidin-25 levels can predict the response to iron supplementation in patients with CKD or requiring HD. A previous study demonstrated that serum levels of hepcidin-25 were not predictive of response to intravenous iron supplementation in HD patients [13]. In contrast, high serum levels of hepcidin-25 have been associated with unresponsiveness to iron supplementation in patients with IDA [21]. In addition, low serum levels of hepcidin-25 have been shown to be predictive of the response to intravenous iron supplementation in patients with CKD not on dialysis [22]. Thus, serum hepcidin-25 may predict the response to iron supplementation in HD patients.

Currently, it is not established whether or not OIT is beneficial in the treatment of IDA associated with HD patients. Similarly, the clinical factors predictive of OIT response are not defined. We hypothesized that the response to OIT depends on the severity of iron depletion in HD patients with IDA. The present study was undertaken to assess the benefit of OIT in HD patients with IDA and to determine whether low levels of serum hepcidin-25, MCV, and serum ferritin predict the response to OIT in these patients.

## 2. Experimental Section

### 2.1. Patient Population

The Institutional Review Board at Public Central Hospital of Matto Ishikawa approved the study on 8 December 2011 (approval code: 23-27) and informed consent was obtained from all patients. During the 3 months before the start of the study, 90 consecutive patients were managed on maintenance HD at our institution. These patients were screened based on the inclusion and exclusion criteria of the study. The inclusion criterion was diagnosis of IDA, which was defined as low levels of Hb (<12 g/dL) and serum ferritin (<100 ng/mL), as described elsewhere [8,23]. Exclusion criteria included hepatic disease, infections, inflammation, gastrointestinal bleeding, cancer, and deficiency of vitamin B_12_ or folate. After the first screening, 51 (56.7%, 34 males) of the 90 HD patients fulfilled the criteria and were enrolled in the study. Of these, there were 50 patients undergoing HD through an arteriovenous fistula and one undergoing HD through an arteriovenous graft. All patients were maintained on HD using the ultrapure dialysate to minimize inflammation [5]. None of the patients received concurrent immunosuppressive agents for underlying kidney disease.

### 2.2. Study Design

In all patients, iron supplementation was withheld for at least 3 months before the study. To examine the benefit of OIT, an 8-week course of oral ferrous fumarate (50 mg/day) was given to 51 HD patients. The dose of ferrous fumarate was chosen based on previous information [24]. All patients received a continuous erythropoietin receptor activator (CERA; methoxy polyethylene glycol-epoetin-β), for 3 months before and during the study period. The dose of CERA was not changed during the entire study period. Patients showing an increase in Hb ≥2 g/dL above baseline after OIT were considered responders, whereas those with a smaller or no change in Hb after OIT were considered non-responders [8].

To determine the influence of HD, nutritional status, calcium/phosphorus status, and comorbidities on patient response to OIT, data on HD vintage, spKt/V, body mass index, serum levels of albumin, prealbumin, calcium, phosphorus, and intact-parathyroid hormone (i-PTH) were measured before OIT. In addition, the presence or absence of any comorbidities, including diabetes mellitus, hypertension, coronary artery disease, congestive heart failure, and vascular disease, were identified before OIT. All data were compared between the responder group and the non-responder group.

To examine whether hepcidin-25, MCV, and ferritin are useful biomarkers of iron storage status, the correlation between hepcidin-25, MCV, ferritin, and baseline Hb before OIT was analyzed. To determine whether hepcidin-25, MCV, and ferritin predict OIT response, the correlation between these variables and ΔHb after OIT was analyzed. To determine the influence of inflammation on OIT response, the levels of C-reactive protein (CRP) were compared between the groups, and the correlation between CRP, hepcidin-25, ferritin and MCV was analyzed. For the purpose of the study, MCV, serum levels of hepcidin-25, iron, total iron binding capacity (TIBC), transferrin, transferrin saturation (TSAT), and CRP were measured before OIT. Serum levels of ferritin and baseline Hb were measured at 0 and 3 months before OIT. The change in Hb (ΔHb) from the baseline was measured after completion of OIT.

Finally, the predictive factors of OIT response were determined by a multiple linear regression analysis and a stepwise multiple linear regression analysis. The values of the variables for predicting OIT response were further analyzed by a receiver operating characteristics (ROC) curve analysis [13,23], and the optimal cutoff values of hepcidin-25, MCV, and ferritin for discriminating the responders from the non-responders were calculated.

### 2.3. Measurements of Parameters

All samples were drawn from the dialysis access just before the start of dialysis. The samples were then centrifuged, and aliquots of sera were immediately stored at −80 °C. Hb, MCV, and serum levels of albumin, prealbumin, calcium, and phosphorus were measured with a conventional autoanalyzer. Serum albumin was determined by the modified bromocresol purple method, and CRP was determined by turbidimetric immunoassay. Serum iron and TIBC were measured by nitroso-PSAP chromogenic method. Serum transferrin was measured by an immunoturbidimetric assay, and i-PTH was determined by electrochemiluminescence immunoassay. TSAT (%) was calculated by the formula

(serum iron/TIBC) × 100.(1)

Serum hepcidin-25 was measured using a high-throughput liquid chromatography tandem-mass spectrometry (LC-MS/MS) method, as described in our previous study [19].

### 2.4. Statistical Analysis

Data are expressed as mean ± SD. Comparison of means was performed using the Mann-Whitney *U* test, and that of two proportions was performed using the Fisher’s exact test. Correlation between parameters was assessed using the Pearson correlation coefficient. A multiple linear regression analysis and a stepwise multiple linear regression analysis were performed to evaluate the contribution of multiple confounding factors to the response to OIT. A ROC curve analysis was further performed to determine significant predictors of the response to OIT. Area under the ROC curves (AUCs) for hepcidin-25, MCV, ferritin, iron, TIBC, and transferrin were calculated as an index of predictive power of OIT response. An AUC of >0.7 was considered significant. The variability of sensitivity/specificity estimates was indicated by 95% exact confidence intervals (CIs). The predictive power of the various ROC AUCs was compared using the method of DeLong *et al.* [25]. A software package (SPSS, version 20.0; SPSS Inc., Chicago, IL, USA) was used for statistical analysis. A *p* value < 0.05 was considered significant.

## 3. Results

**Figure 1:**
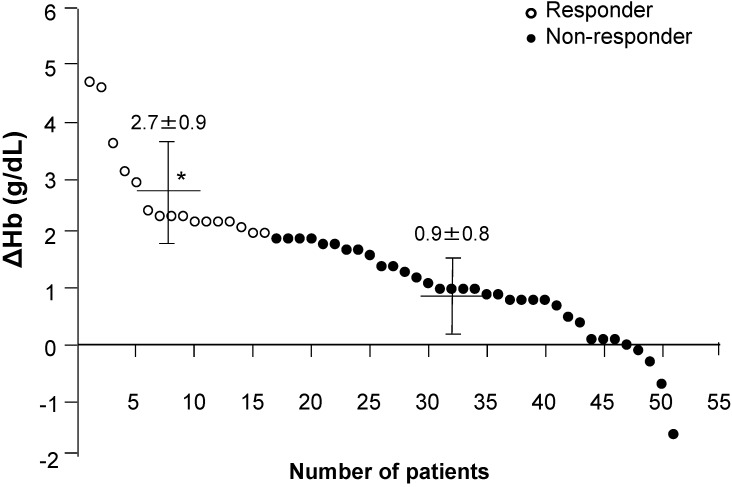
Distribution of ΔHb after oral iron therapy (OIT) across the total HD study population. The ΔHb after OIT was significantly higher in the responder group (2.7 ± 0.9 g/dL, *n* = 16) compared to the non-responder group (0.9 ± 0.8 g/dL, *n* = 35). Bars indicate mean ± SD. * *p* < 0.01, *vs.* non-responder group. ΔHb, change in Hb after OIT from baseline.

The mean level of Hb for all study patients (*n* = 51) increased from 9.7 ± 1.4 g/dL at baseline to 11.1 ± 1.3 g/dL (*p* < 0.01) after OIT. After 8 weeks of OIT, there were 16 responders and 35 non-responders, respectively. The mean change in Hb level (ΔHb) after OIT was significantly higher in the responder group (2.7 ± 0.9 g/dL, *p* < 0.01) compared to the non-responder group (0.9 ± 0.8 g/dL, Figure 1).

Table 1 summarizes the baseline patient characteristics before OIT. There was no significant difference between the responders and non-responders with regard to age at the time of the study, gender ratio, body mass index, HD vintage, spKt/V, laboratory data for nutritional status, calcium/phosphorus, i-PTH, and CRP. None of the patients had positive CRP (>8.0 mg/L) [26], suggestive of an absence of significant inflammation. No difference was found in the dose of CERA and the rate of comorbidities between the groups.

Table 2 shows the data for Hb, serum levels of hepcidin-25, MCV, and iron-related parameters before and at the start of OIT between the responders and non-responders. The mean levels of Hb 3 months before OIT and at the start of the study were lower in the responder group (*p* < 0.05, respectively) than in the non-responder group. In the responder group, the mean levels of Hb at the start of the study were significantly lower than those 3 months before OIT, whereas the levels of Hb did not differ at these time points in the non-responder group. Serum levels of hepcidin-25 and MCV were significantly lower in the responder group (*p* < 0.05, respectively) than in the non-responder group.

**Table 1:** Baseline characteristics of the study population.

Clinical Characteristics	Responder Group (*n* = 16)	Non-Responder Group (*n* = 35)	*p*-Value
Age (years)	61.0 ± 14.1	75.8 ± 9.2	0.054
Female (%)	39.0	31.0	0.798
Body mass index (kg/m^2^)	21.8 ± 3.6	20.9 ± 2.5	0.859
HD vintage (years)	10.3 ± 9.2	6.9 ± 6.9	0.931
spKt/V	1.48 ± 0.35	1.36 ± 0.30	0.884
Serum albumin (g/dL)	3.4 ± 0.4	3.4 ± 0.4	0.578
Serum prealbumin (mg/dL)	29.1 ± 6.5	30.5 ± 9.3	0.288
Serum calcium (mg/dL)	9.3 ± 1.1	9.1 ± 0.8	0.739
Serum phosphorus (mg/dL)	5.3 ± 1.7	5.7 ± 1.5	0.183
i-PTH (pg/mL)	107.9 ± 102.4	93.9 ± 79.6	0.701
CRP (mg/L)	0.97 ± 0.65	1.62 ± 2.34	0.142
CERA dose (IU/kg/week)	0.95 ± 0.52	0.77 ± 0.37	0.885
*Comorbidities (%)*			
Diabetes mellitus	43.8	22.9	0.129
Hypertension	81.3	88.6	0.684
Coronary artery disease	56.3	45.7	0.485
Congestive heart failure	18.8	14.3	0.684
Vascular disease	56.3	40.0	0.279

Data are expressed as mean ± SD, unless otherwise indicated. CERA, continuous erythropoietin receptor activator (methoxy polyethylene glycol-epoetin-β); CRP, C-reactive protein; HD, hemodialysis; i-PTH, intact-parathyroid hormone.

**Table 2:** Hb, serum hepcidin-25, MCV and iron-related parameters before the start of OIT.

Variable	Responder Group (*n* = 16)	Non-Responder Group (*n* = 35)
Hb 3 months before study (g/dL)	9.5 ± 0.9 *	10.3 ± 1.3
Hb at start of study (g/dL)	8.8 ± 1.2 * ^#^	10.1 ± 1.3
Serum hepcidin-25 (ng/mL)	10.8 ± 16.7 *	32.8 ± 38.3
MCV (fL)	85.1 ± 5.3 *	89.2 ± 3.7
Serum ferritin 3 months before study (ng/mL)	19.8 ± 12.3	30.6 ± 30.4
Serum ferritin at start of study (ng/mL)	17.7 ± 17.5 *	33.5 ± 24.2
Serum iron (μg/dL)	67.3 ± 21.3	77.8 ± 28.7
TIBC (μg/dL)	307.8 ± 59.2	278.7 ± 42.3
Serum transferrin (mg/dL)	239.8 ± 49.0 *	214.2 ± 38.3
TSAT (%)	22.9 ± 8.6 *	30.0 ± 14.7

Data are expressed as mean ± SD. * *p* < 0.05, *vs.* non-responder group, ^#^ *p* < 0.05, *vs.* the data obtained at 3 months before the study. Hb, hemoglobin; MCV, mean corpuscular volume; TIBC, total iron-binding capacity; TSAT, transferrin saturation.

None of the patients had microcytosis (MCV < 80 fL) or macrocytosis (MCV > 100 fL) [7]. Serum levels of ferritin at the start of OIT were significantly lower in the responders (*p* < 0.05) than in the non-responders. Serum levels of ferritin at the start of the study in the responders tended to be lower than those 3 months before the study, whereas they did not differ in the non-responders. Serum levels of iron tended to be lower, and TIBC tended to be higher in the responders than in the non-responders, but this did not reach a statistical significance. Serum levels of transferrin were higher (*p* < 0.05) and TSAT was lower in the responder group (*p* < 0.05) than in the non-responder group.

Table 3 shows the correlation between serum hepcidin-25, MCV, serum ferritin, and baseline Hb before and ΔHb after OIT. Serum levels of hepcidin-25 were positively correlated with those of ferritin (*r* = 0.86, *p* = 0.0001). The levels of serum hepcidin-25 (*r* = 0.44, *p* = 0.0006), MCV (*r* = 0.30, *p* = 0.0167), and serum ferritin (*r* = 0.51, *p* = 0.0001) were positively correlated with those of baseline Hb before OIT. No significant correlation was found between MCV and either serum hepcidin-25 or ferritin. In contrast, ΔHb after OIT was negatively correlated with serum hepcidin-25 (*r* = −0.41, *p* = 0.0016), MCV (*r* = −0.41, *p* = 0.0013) and serum ferritin (*r* = −0.42, *p* = 0.001).

**Table 3:** Correlation between serum hepcidin-25, MCV, serum ferritin and baseline Hb before and ΔHb after OIT.

Variable	R	*p*-Value	95% CI
Serum hepcidin-25 and ferritin	0.86	0.0001 *	[0.77, 0.92]
Serum hepcidin-25 and baseline Hb	0.44	0.0006 *	[0.19, 0.64]
Serum hepcidin-25 and MCV	0.20	0.1655	[−0.08, 0.45]
MCV and baseline Hb	0.30	0.0167 *	[0.02, 0.53]
MCV and serum ferritin	0.05	0.3512	[−0.22, 0.33]
Serum ferritin and baseline Hb	0.51	0.0001 *	[0.27, 0.69]
Serum hepcidin-25 and ΔHb	−0.41	0.0016 *	[−0.61, −0.15]
MCV and ΔHb	−0.41	0.0013 *	[−0.61, 10.15]
Serum ferritin and ΔHb	−0.42	0.0010 *	[−0.63, −0.17]

Correlation between parameters was analyzed using the Pearson correlation coefficient. * Statistically significant. Hb, hemoglobin; ΔHb, change in Hb after OIT from baseline; MCV, mean corpuscular volume.

**Figure 2:**
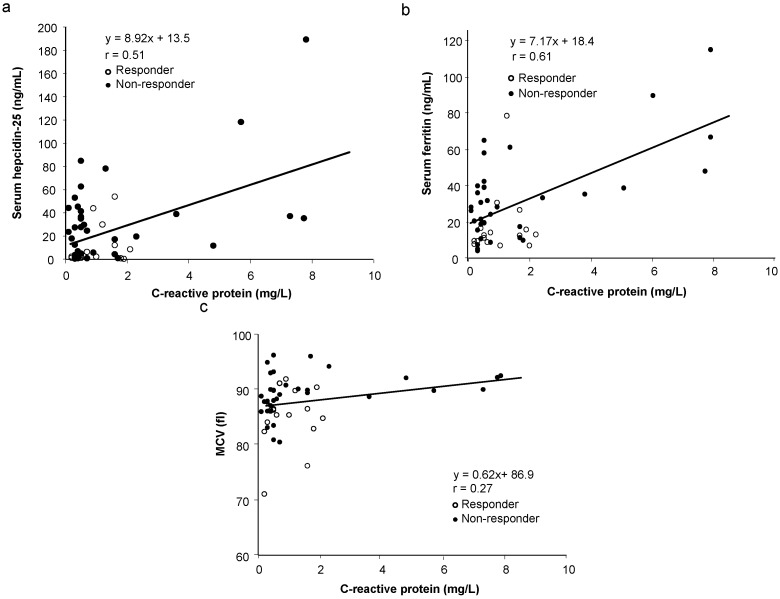
Correlation between serum levels of CRP with baseline levels of serum hepcidin-25, ferritin, and MCV before OIT in responders and non-responders. There is a significant and positive correlation between CRP and serum hepcidin-25 (Figure 2a, *r* = 0.51, *p* < 0.01) as well as serum ferritin (Figure 2b, *r* = 0.61, *p* < 0.01). However, no correlation was found between CRP and MCV (Figure 2c, *r* = 0.27, *p* = 0.97).

Since inflammation could influence serum levels of hepcidin-25 and ferritin [7,9,10,11,15,16], thereby affecting OIT response, the correlation between CRP and serum hepcidin-25, ferritin, or MCV was analyzed. Surprisingly, despite normal levels of CRP in all patients, there was a significant and positive correlation between CRP and serum hepcidin-25 (Figure 2a, *r* = 0.51, *p* < 0.01) as well as serum ferritin (Figure 2b, *r* = 0.61, *p* < 0.01). However, no significant correlation was found between CRP and MCV (Figure 2c, *r* = 0.27, *p* = 0.97).

We examined the contribution of several variables to OIT response in HD patients with IDA. A multiple linear regression analysis showed that none of the variables used in the analysis could predict OIT response (Table 4). However, a stepwise multiple linear regression analysis revealed that serum hepcidin-25, MCV, and baseline Hb before OIT could predict OIT response in HD patients (Table 5).

**Table 4:** Contribution of variables to OIT response with ΔHb increment evaluated by multiple linear regression analysis.

Variable	Regression Coefficient	*p*-Value	95% CI
Age	0.001	0.920	[−1.329, 0.462]
Gender	0.001	0.998	[−0.717, 0.719]
DM or non DM	−0.433	0.333	[−1.329, 0.462]
Serum hepcidin-25	−0.008	0.338	[−0.026, 0.009]
Baseline Hb	−0.146	0.466	[−0.550, 0.257]
MCV	−0.071	0.107	[−0.159, −0.016]
Serum ferritin	−0.004	0.786	[−0.037, 0.028]
Serum iron	0.004	0.546	[0.010, 0.018]
Serum transferrin	0.007	0.671	[−0.027, 0.041]
CRP	1.095	0.289	[−0.967, 0.157]
CERA dose	0.343	0.487	[−0.647, 1.334]

CERA, continuous erythropoietin receptor activator (methoxy polyethylene glycol-epoetin-β); CI, confidence interval; CRP, C-reactive protein; DM, diabetes mellitus; Hb, hemoglobin; ΔHb, change in Hb after OIT from baseline; MCV, mean corpuscular volume.

**Table 5:** Contribution of variables * to OIT response with ΔHb increment evaluated by stepwise multiple linear regression analysis.

Variable	Regression Co-Efficient	F-value
Serum hepcidin-25	−0.00750	2.711 **
MCV	−0.06538	3.652 **
Baseline Hb	−0.27063	4.388 **

* Variables included in the analysis were: serum hepcidin-25, MCV, baseline Hb, serum levels of ferritin, iron, TIBC, TSAT, transferrin, CRP, and CERA dose. ** Statistically significant.

**Figure 3:**
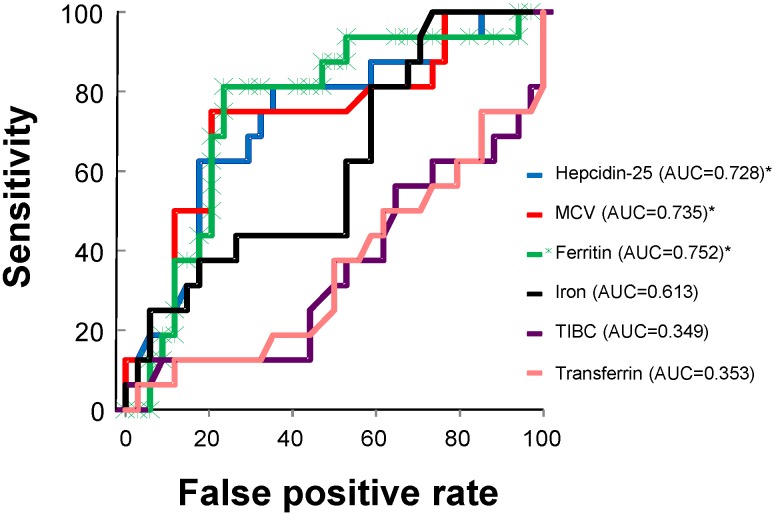
A ROC curve analysis for the evaluation of variables predictive of the response to OIT. A ROC curve analysis revealed that serum hepcidin-25 (95% CI = 0.576–0.880, *p* = 0.001), MCV (95% CI = 0.580–0.891, *p* = 0.008), and serum ferritin (95% CI = 0.605–0.899, *p* = 0.004), but not serum iron (95% CI = 0.448–0.778, *p* = 0.201), TIBC (95% CI = 0.178–0.520, *p* = 0.088), or serum transferrin (95% CI = 0.183–0.523, *p* = 0.096), emerged as significant predictors of the response to OIT. * Statistically significant. AUC, area under the curve; MCV, mean corpuscular volume; TIBC, total iron binding capacity.

The predictive power of the variables used in the analysis for the response to OIT was further evaluated by a ROC curve analysis. Using a ROC curve analysis, serum hepcidin-25 (AUC = 0.728, 95% CI = 0.576–0.880, *p* = 0.001), MCV (AUC = 0.735, 95% CI = 0.580–0.891, *p* = 0.008), and serum ferritin (AUC = 0.752, 95% CI = 0.605–0.899, *p* = 0.004) emerged as significant predictors of the response to OIT (Figure 3). Serum iron (AUC = 0.613, 95% CI = 0.448–0.778, *p* = 0.201), TIBC (AUC = 0.349, 95% CI = 0.178–0.520, *p* = 0.088), and serum transferrin (AUC = 0.353, 95% CI = 0.183–0.523, *p* = 0.096) could not predict the response to OIT. The combination of serum hepcidin-25, MCV, and serum ferritin seemed to have more predictive power for the response to OIT (AUC = 0.824, 95% CI = 0.693–0.954) compared to that of each variable alone. In addition, the ROC curve analysis revealed that the optimal cut-off values of serum hepcidin-25, MCV, and serum ferritin for distinguishing the responders from the non-responders were 12.5 ng/mL (sensitivity 81.3%, specificity 61.8%), 87.5 fL (sensitivity 75.0%, specificity 73.5%), and 16.7 ng/mL (sensitivity 81.3%, specificity 76.5%), respectively.

Adverse effects attributable to OIT included two cases of mild and transient nausea, whereas no other adverse effects were noted. None of the patients discontinued OIT. There was good adherence to taking the oral iron medication in our study population.

## 4. Discussion

Our study suggests that serum hepcidin-25, MCV, and serum ferritin could be useful markers of iron storage status and predictive of the response to OIT, and that the benefit of OIT may be dependent on the severity of IDA in HD patients.

Two independent research groups emphasized that serum hepcidin-25 is not a reliable guide for the use of iron or erythropoietin-stimulating agent (ESA) therapy because of its considerable variability in HD patients [27,28]. In both studies, serum hepsicin-25 was measured using enzyme-linked immunoassay (ELISA) and/or mass spectrometry, and a majority of the patients had high CRP levels, indicative of significant inflammation, which increases hepcidin-25. The data for serum levels of hepcidin-25 measured by mass spectrometry are generally lower than those measured by ELISA because the latter method also detects the non-bioactive hepcidin isomers [27,28]. In one study, the variability of serum hepcidin-25 was still high in a small number of HD patients with normal CRP levels [27]. However, another study suggested that the variability of serum hepcidin-25 levels is dependent on fluctuations in the inflammatory state [28]. The LC-MS/MS method for the measurement of serum hepcidin-25 used in our study is more accurate than ELISA [29], and none of our patients had elevated CRP, indicative of an absence of significant inflammation. Thus, the data for serum hepcidin-25 in our study support its use as an index of iron metabolism.

In our series, we found no statistical differences in the demographic data before OIT between the responders and the non-responders. However, because of the small sample size in our study, these data may be underpowered to detect a statistical difference in the variables analyzed between the groups.

In the present study, there was a positive correlation between baseline Hb before OIT and either serum hepcidin-25, MCV, or serum ferritin, and between serum hepcidin-25 and ferritin, as described in previous studies [13,14,15,16,17,18]. Prohepcidin has also been shown to be positively correlated with hematocrit in HD patients [30]. Our data, together with these data, suggest that serum hepcidin-25, MCV, and serum ferritin could be useful markers of iron storage status, as the levels of hepcidin-25 and ferritin are mainly modulated by iron stores [10,31]. Our finding that hepcidin-25, MCV, and ferritin reflect iron storage status may be applicable to a patient population in which patients with overt inflammation are excluded, as in our study population. However, these findings may not be applicable to HD patients in general, as these patients frequently have high levels of inflammation, which increases serum levels of hepcidin-25.

Previous studies suggested that MCV correlated positively with serum ferritin [32,33], and both MCV and serum ferritin were decreased after cessation of iron therapy in HD patients [34], suggesting that an alteration of MCV may occur in parallel with that of serum ferritin. However, we found no correlation between MCV and either serum hepcidin-25 or ferritin, whereas there was a positive correlation between serum hepcidin-25 and ferritin in our study, suggesting that serum hepcidin-25, but not MCV, is a major predictor of serum ferritin levels in HD patients [15]. In support of our finding, previous reports have shown no correlation between MCV and serum ferritin in IDA patients requiring HD [35,36] or those without kidney disease [6]. Our finding that serum hepcidin-25, but not MCV, is a predictor of serum ferritin levels in HD patients may be explained by the fact that an alteration of serum hepcidin-25 or ferritin occurs more rapidly than changes in MCV, since prolonged iron-deficient erythropoiesis is needed to change MCV [6]. In fact, up to 40% of non-HD patients with IDA have normal MCV, indicating that normal MCV does not rule out IDA [7].

Regarding predictive variables for response to OIT in HD patients with IDA, we found a negative correlation between ΔHb after OIT and serum hepcidin-25, MCV, or serum ferritin, suggesting that hepcidin-25, MCV, and ferritin may independently predict the response to OIT. Although a multiple linear regression analysis failed to show the predictive values of these variables, a stepwise multiple regression analysis revealed that serum hepcidin-25, MCV, and baseline Hb before OIT could predict the response to OIT. This is further supported by the ROC curve analysis, showing that serum hepcidin-25, MCV, and serum ferritin could predict OIT response, and that these variables, when combined, have greater predictive value for the response to OIT as compared to the predictive value of each variable alone. In support of our finding, previous studies have shown the predictive value of low serum hepcidin-25 for the good response to intravenous iron supplementation in CKD patients not on dialysis [22]. In addition, HD patients with high levels of serum ferritin [37,38] and non-HD patients with high levels of serum hepcidin-25 [21] have been resistant to intravenous or oral iron supplementation. In contrast, other reports showed that serum hepcidin-25 was of minimal value in predicting response to intravenous iron supplementation in HD patients [13]. These contradictory results may be due to the difference in the patients’ background, the presence or absence of inflammation, HD vintage, a different method for HD with or without the use of ultrapure dialysate, the dose and the method of iron supplementation (intravenous *versus* oral), iron depletion or repletion status, and different ESA regimens. It is noteworthy that the response to OIT observed in our patients is likely because the dose of ferrous fumarate used is adequate to increase iron bioavailability for erythropoiesis [39], while all other potential confounding variables remained stable throughout the study.

Serum levels of hepcidin-25 may predict the response to OIT not only in patients with HD or CKD but also in those without kidney disease and IDA. Hepcidin-25 could predict iron bioavailability from ferrous fumarate in normal healthy volunteers [39]. Moreover, patients with anemia may be classified as iron-responsive and non-responsive based on hepcidin-25 expression, thus guiding the use of iron supplementation in those who are likely to benefit the most [40]. The value of serum hepcidin-25 for predicting response to OIT has also been shown in patients with IDA [21].

In the case of severe iron depletion, low levels of hepcidin-25 can enhance iron absorption and efflux, thereby increasing iron availability for erythropoiesis [10]. This may account for the benefit of OIT in HD patients with low levels of serum hepcidin-25, MCV, and serum ferritin. In support of this idea, intravenous iron supplementation has been shown to be effective in HD patients with low levels of serum ferritin, and the magnitude of response and the proportion of patients responding have been shown to be correlated with the percentage of hypochromic erythrocytes before intravenous iron supplementation [13]. In contrast, HD patients with normal levels of serum hepcidin-25 and ferritin failed to respond to OIT in our study, suggesting that iron absorption and efflux may be inhibited, thereby reducing iron availability for erythropoiesis in HD patients with normal levels of serum hepcidin-25 and ferritin [31]. This finding has been supported by the following observations: (1) high levels of serum hepcidin-25 and ferritin have been associated with nonresponsiveness to OIT in non-HD patients [21]; (2) HD patients with high levels of serum ferritin have been resistant to iron supplementation and thus required high dose of iron [37,38]; and (3) HD patients with normal MCV have failed to respond to intravenous iron supplementation [32].

Inflammation could enhance resistance to iron supplementation in HD patients [38,41] because it increases hepcidin-25 and ferritin [7,9,10]. In fact, serum levels of hepcidin-25 have been shown to be positively correlated with CRP levels in patients with CKD [22] and those with HD [16,28]. Since none of our patients had elevated CRP, absence of inflammation may be crucial for OIT response in HD patients with IDA. Furthermore, there was a significant and positive correlation between CRP, serum hepcidin-25, and ferritin in the absence of inflammation in our study. Since the liver is the site of hepcidin-25 and CRP production as well as the storage of ferritin [9,10], our data suggest that hepatic sensing of low levels of serum hepcidin-25 and ferritin may trigger the mechanism by which iron availability for erythropoiesis is increased [10], leading to a better response to OIT in certain HD patients with minor inflammation.

It remains unclear whether MCV predicts the response to iron therapy in patients requiring HD or with CKD. We found that MCV alone, or together with serum hepcidin-25 and ferritin, could be predictive of the response to OIT in HD patients. In other clinical settings, MCV has little value in predicting the OIT response of IDA patients without kidney disease [42], whereas the combination of low levels of MCV and serum ferritin could predict the response to OIT in anemic patients with rheumatoid arthritis [43]. Further investigation would be necessary to determine the predictive value of MCV for OIT response in HD patients.

Gastrointestinal adverse effects are more common with OIT than with intravenous iron supplementation, whereas serious adverse effects such as hypotension and anaphylaxis are more common with intravenous iron supplementation [1,7]. Limitation of OIT as the treatment of IDA has been emphasized because of its gastrointestinal adverse effects, poor patient’s adherence, poor intestinal absorption, and low efficacy [7,44]. Our study, however, shows minor OIT-related adverse effects and good compliance, probably because of the low dose of ferrous fumarate. Thus, OIT can be beneficial in certain HD patients with IDA.

## 5. Conclusions

Our study suggests that serum hepcidin-25, MCV, and serum ferritin could be useful markers of iron storage status and predictors of OIT response in HD patients with IDA. The beneficial effect of OIT may be dependent on the severity of IDA in these patients. Further studies with a larger number of HD patients are necessary to confirm these findings.
